# Forest management optimization across spatial scales to reconcile economic and conservation objectives

**DOI:** 10.1371/journal.pone.0218213

**Published:** 2019-06-10

**Authors:** Tähti Pohjanmies, Kyle Eyvindson, Mikko Mönkkönen

**Affiliations:** 1 University of Jyvaskyla, Department of Biological and Environmental Sciences, Jyväskylä, Finland; 2 School of Resource Wisdom, University of Jyväskylä, Jyväskylä, Finland; Albert-Ludwigs-Universitat Freiburg, GERMANY

## Abstract

Conflicts between biodiversity conservation and resource production can be mitigated by multi-objective management planning. Optimizing management for multiple objectives over larger land areas likely entails trading off the practicability of the process against the goodness of the solution. It is therefore worthwhile to resolve how large areas are required as management planning regions to reconcile conflicting objectives as effectively as possible. We aimed to reveal how the extent of forestry planning regions impacts the potential to mitigate a forestry-conservation conflict in Finland, represented as a trade-off between harvest income and deadwood availability. We used forecasted data from a forest simulator, a hierarchy of forestry planning regions, and an optimization model to explore the production possibility frontier between harvest income and deadwood. We compared the overall outcomes when management was optimized within the different-sized planning regions in terms of the two objectives, the spatial variation of deadwood, and the optimal combinations of management regimes. Increasing the size of the planning regions did produce higher simultaneous levels of the two objectives, but the differences were most often of the magnitude of only a few percentages. The differences among the scales were minor also in terms of the spatial variation in deadwood availability and in the optimal management combinations. The conflict between timber harvesting and deadwood availability is only marginally easier to mitigate at large spatial scales than at small forest ownership scales. However, regardless of the spatial scale of planning, the achievable solutions may not be good enough to safeguard deadwood-dependent biodiversity without active deadwood creation.

## Introduction

Logging is one of the main causes of biodiversity loss in forest environments [1]. Intensive management and harvesting of forests modify their structure at multiple spatial scales, thereby affecting the availability of habitats and resources to wildlife [2–4]. Logging may negatively affect forest biodiversity even in areas where widespread forest loss is not taking place and that are characterized by net gains in growing forest stock, such as in the boreal forests of Finland [5]. In Finland, intensive and widespread forestry has substantially altered forest ecosystems as compared to natural forests in terms of stand age distribution, stand structure, tree species distribution, microclimatic conditions, and the amount of deadwood in the forest [6,7]. The consequences have included an increase in the amount of harvestable timber on one hand, and habitat degradation and endangerment of forest species on the other hand [8,9]. Currently, pressures to further intensify forest use continue [10] at the same time as concerns for the preservation of forest biodiversity escalate [11], maintaining challenging conflicts between economic and conservation objectives. It is a situation that recurs throughout the world in different kinds of production ecosystems [12].

Conflicts between resource production and conservation may be mitigated by adopting management practices that are designed to support both economic objectives and biodiversity. Management solutions that balance conflicting objectives can be sought at multiple spatial scales. In forest management, stand-level options that are considered comparatively biodiversity-friendly include mixed-species forestry, extended rotation times, retention forestry, and continuous cover forestry [3,13–15]. At the landscape level, land-use optimization may be used for effective protected area prioritization [16], cost-effective conservation planning [17], as well as to identify the optimal spatial allocation of management actions [18]. Most likely a combination of strategies is necessary: for example, in Finland where only a small proportion of forest area is protected, preservation of forest biodiversity likely requires actions in commercially utilized forests in addition to the increased protection of forests [7,19].

Forest stands may respond to management activities differently due to factors such as silvicultural history, species composition, and site fertility [15]. Different management regimes may therefore support desired biodiversity features as well as timber production to an extent that varies among stands [20]. As a result, when targeting a compromise between conflicting biodiversity conservation and economic objectives, the optimal forest management plan may consist of a combination of alternative regimes applied over the target area [21,22]. Such optimal management plans may be found by applying multi-objective optimization: the management alternatives, their outcomes in terms of the multiple objectives, and applicable constraints are integrated into an optimization problem, which is solved using mathematical methods [23]. Theoretically, the more alternative management regimes are available, the larger is the area over which they are applied, and the higher is its resolution, the larger is the number of possible solutions, and the better is the ability of the process to find solutions that are good in terms of all objectives. However, increasing the number of available options increases the computational complexity of the optimization problem. In addition, expanding the size of the target area in particular may hinder the practicality and acceptability of the identified plan if areas spanning over more than a single ownership are involved [24]. There is variation among forest owners with respect to their values and objectives as well as to their opportunities to actualize their preferences into management decisions [25]. If management planning is conducted at a scale comprising multiple ownerships, it may be necessary to integrate owner-specific objectives into the decision-making process to improve the plan’s acceptability [26]. However, this unavoidably constraints the available solutions. It is thus of interest to find out how small planning regions can be in order to get the most use out of multi-objective optimization in management planning.

In an earlier study [27] we showed that the effectiveness of forest management optimization to promote the co-production of carbon storage and income from timber harvests in Finland is positively affected by the size of the planning region, but only marginally beyond relatively small areas (approximately 200 ha). In this article, we tackle the question of how the spatial scale of management optimization affects the capability to mitigate the conflict between timber harvesting and deadwood availability. Deadwood availability, both in terms of volume and diversity, is substantially lower in managed than natural forests: the average volume of deadwood in managed forests in Finland is about 5 m^3^/ha [28], while in natural old-growth forests it can be more than an order of magnitude higher (60–90 m^3^/ha) [6]. Lack of deadwood resources is underlying the endangerment of many forest species [6,8]. Increasing deadwood availability at the stand level can be achieved through active restoration [29] or by applying stand management that promotes the formation of deadwood (e.g. refraining from thinnings) [22,30]. Increasing the amount and diversity of deadwood resources in forests is the first step towards promoting deadwood-dependent biodiversity [31,32]. Landscape-level planning has been widely utilized in land-use optimization to maximize conservation benefits while minimizing economic or opportunity costs [16,18,33,34]. In particular, landscape-level forest management optimization has been shown to enable improvements in deadwood availability with comparatively small economic costs [22]. Here, we aim to reveal what is the sufficient spatial scale to achieve these improvements.

The questions we aim to answer are: 1) Does land-use optimization over larger spatial scales lead to more efficient mitigation of the forestry-conservation conflict of timber harvesting vs. deadwood availability? 2) How does increasing the spatial scale of land-use optimization affect the spatial variation of deadwood availability in the forest? Correspondingly, we hypothesize that 1) land-use optimization over larger areas leads to higher simultaneous levels of timber harvesting and deadwood availability, but 2) the variation in deadwood availability also increases as the spatial scale is increased. To resolve the study questions, we simulate the development of nearly 29,000 forest stands in Finland under a range of alternative stand management regimes, optimize the allocation of the regimes within planning regions of increasing size to maximize the simultaneous production of economic value and deadwood resources, and compare the outcomes across scales.

## Methods

### Forest data and forest growth simulations to predict two objectives

**Fig 1:**
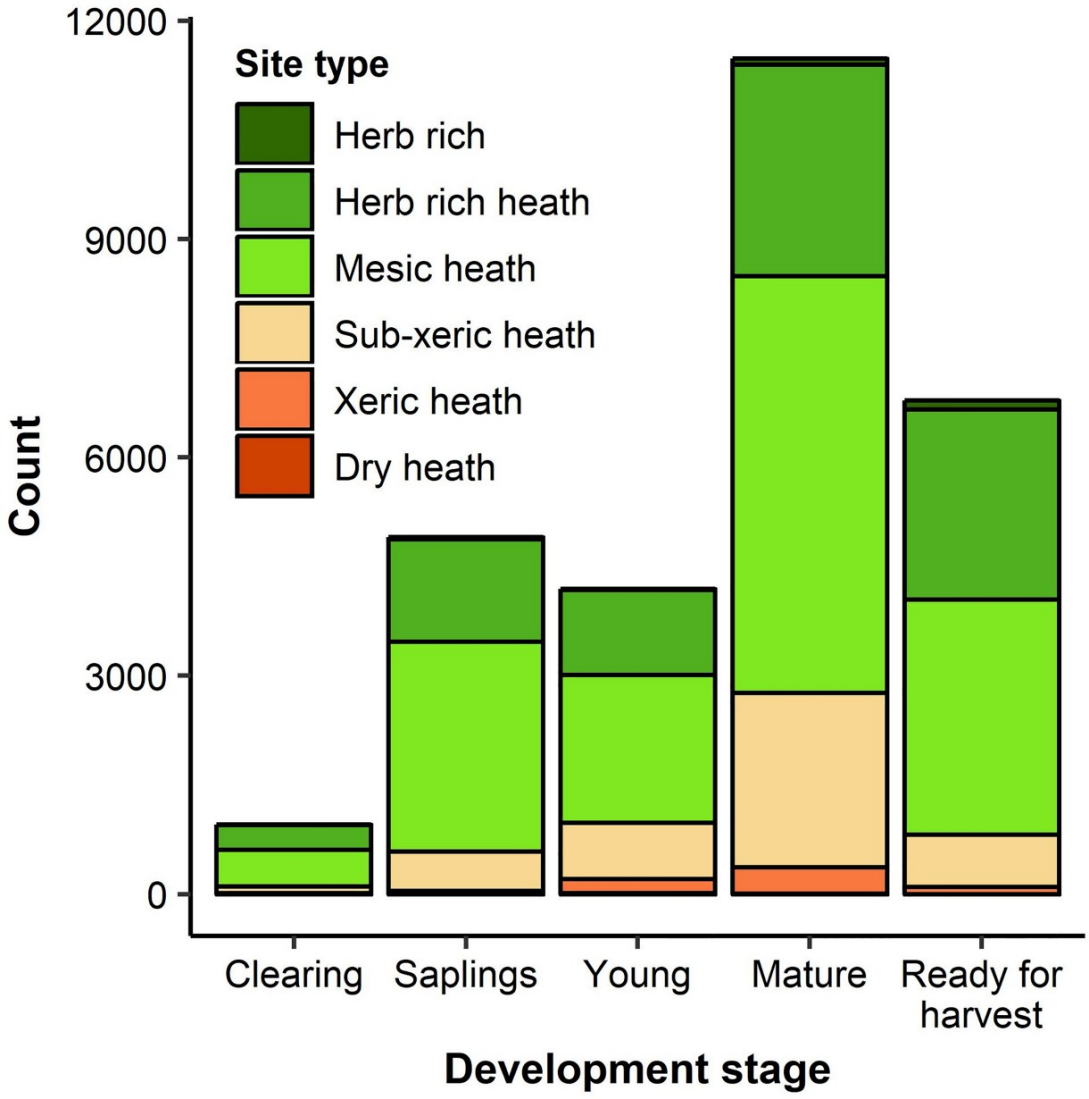
Characteristics of the stands comprising the study area. Distribution of the forest stands included in the study classified into present development stages (x axis) and site types (bar fill). The development stages and site types were defined in the forest inventories conducted by the Finnish Forest Centre. ‘Young’ stands have a mean diameter at breast height of 8–16 cm, and ‘Mature’ stands a mean diameter at breast height greater than 16 cm but are not yet ready for final harvest. ‘Ready for harvest’ stands are greater than 16 or 14 m in dominant height and at least greater than 70 or 90 years in age, as defined in the current Finnish recommendations for the timing of final felling [35]. Site types are presented according to the Finnish forest classification system [36] and range from fertile mixed-species stands (herb rich) to less fertile, pine-dominated stands (dry heath).

We used forest inventory data produced by the Finnish Forest Centre from a total of 28,886 forest stands. The stands cover a total area of 43,970 ha and are located in southern and central Finland. The stand-level forest inventory data are produced using remote sensing and field measurements and include basic information of a stand (e.g., location, soil type) and detailed information about its current structure (e.g., tree species distribution, tree density, tree strata). The 28,886 stands used in this study vary in their current condition, illustrated by e.g. their development stage, and forest type with the most common forest types of mesic heaths, herb rich heaths, and sub-xeric heaths (Fig 1).

The future development of the stands was predicted with the forest growth simulator SIMO [37]. SIMO uses forest growth models [38,39] to predict stand development based on the stand’s permanent characteristics and current structure as well as the forestry operations applied. Deadwood dynamics were estimated by combining the deadwood creation predicted by SIMO (based on species-specific models of individual tree mortality [38,39] and the quantification of harvest residues) with a deadwood decomposition model [40]. SIMO can be used to explore the outcomes of alternative stand management choices as the timing and intensity of forestry operations can be adjusted to reflect different forest management regimes. We simulated the development of the stands for one hundred years into the future under a total of 19 alternative management regimes (Table 1). These were: a ‘business-as-usual’ even-aged forestry regime, 16 modified versions of the ‘business-as-usual’ regime, a continuous cover forestry regime, and permanent set-aside. The ‘business-as-usual’ regime consists of one or more thinnings, final felling by clear-cut, and stand regeneration, with the activities timed according to the current Finnish forest management recommendations [35]. In the modified versions of the ‘business-as-usual’ regime, the timing of final felling was postponed, thinnings were omitted, or green tree retention at final felling was increased. The continuous cover forestry regime consisted of regular harvests of the stand’s largest trees down to a predefined minimum basal area. Finally, if the stand was set aside, no forestry operations were conducted and no timber was harvested from the stand. For more details on the management regimes, see Eyvindson et al. [21].

**Table 1:** Management regimes. The alternative management regimes applied in the forest growth simulations. For simplicity, the modified versions of ‘business-as-usual’ management have been grouped into 4 categories based on their defining features (extended rotation time, green tree retention, thinnings before final felling, or no thinnings).

Management regime	Acronym	Description
Business-as-usual	BAU	Even-aged rotation forestry based on current Finnish forest management recommendations. Timing of final felling depends on site type, dominant height, and age, with an average rotation length of 80 years. Final felling is followed by site preparation, planting or seedling trees. Between 1–3 thinnings are conducted during the rotation, depending on site type, dominant height, and basal area. At final felling, 5 trees/ha are left as retention trees.
Extended rotation time	BAU ext	Modified BAU with final felling postponed by 5–30 years.
Green tree retention	BAU w GTR	Modified BAU with either 30 trees/ha or 30m^3^/ha retained at final felling.
Thinnings before final felling	BAU w thin	Modified BAU with thinnings allowed both before and after final felling.
No thinnings	BAU wo thin	Modified BAU with no thinnings allowed.
Continuous cover forestry	CCF	Regular harvests of the stand’s largest trees down to a predefined minimum basal area. Timing of the harvest depends on site type and basal area.
Set-aside	SA	No silvicultural operations, no harvesting.

We used the future stand characteristics and harvest potential predicted by the forest growth simulations to measure two forest management objectives under the alternative management regimes: income from timber harvests and availability of deadwood resources. Harvest income (€) was measured by multiplying the predicted harvests of different timber assortments by their recent average prices [28]. A discount rate of 3% was used to discount income generated in the future. Availability of deadwood resources was measured as the total volume of deadwood (m^3^) multiplied by the diversity of 20 deadwood types (20 combinations of four tree species (Scots pine *Pinus sylvestris*, Norway spruce *Picea abies* and two species of birch, *Betula pendula* and *Betula pubescens*) and five decay stages; decay stages from Mäkinen et al. [40]). We accounted for deadwood diversity in the measure as it has been suggested to be of key importance for species occurrence [32,41]. The diversity of deadwood types was measured with Simpson’s index of diversity. The value of the index varies between 0 and 1 with higher values representing higher diversity. When the total volume of deadwood is multiplied with the diversity index, the resulting value is increased by both deadwood volume and diversity and is the closer to the original value of deadwood volume the higher the deadwood diversity. Harvest income was measured as income generated over the 100-year simulation period discounted to present time and deadwood availability as the predicted availability of deadwood resources averaged across the 100-year simulation period.

### Management optimization at hierarchical spatial scales

We arranged the 28,886 forest stands into groups of increasing size to create hierarchical planning regions. The smallest-scale planning regions, termed “small holdings”, were based on real forest property data and encompassed a mean number of 11 adjacent stands. “Large holdings” were created by grouping together approximately ten adjacent “small holdings” and encompassed a mean number of 127 stands. “Watersheds”, with a mean number of 1,805 stands, corresponded to the actual natural boundaries of third-level catchment areas as defined by the Finnish Environment Institute [42]. The largest scale, “region”, included all 28,886 stands. The mean areas of the planning regions are given in Table 2. The mean size of private forest owners’ holdings in Finland is 30 ha [28], that is, larger than our definition of a “small holding” but smaller than “large holding”.

**Table 2:** Characteristics of planning regions. Total number of groups of stands used as planning regions, mean number of stands in a group, and mean area of a group as defined at the different spatial scales (from Pohjanmies et al. [27]).

Scale	Total number of groups	Mean number of stands	Mean area (ha)
Small holding	2537	11	17.3
Large holding	228	127	192.9
Watershed	16	1805	2748.1
Region	1	28886	43970.2

Stand management was optimized over the planning regions with the aim of reconciling the objectives of harvest income and deadwood availability as well as possible. This was done by allocating the alternative management regimes across the stands in a planning region so that the total levels of both objectives were simultaneously maximized. Specifically, we identified management solutions (allocations of regimes across stands) that maximized deadwood availability with the constraint that harvest income remained above a set required level. These required levels ranged from 0% to 100% of the achievable maximal value of harvest income. By varying the level of the income requirement, we were able to construct the entire production possibility frontier for the two objectives. The exact formulation of the optimization problem was

$$maximize\sum_{i=1}^{n}x_{i,j}$$

$$subject\;to\sum_{i=1}^{n}y_{i,j}\geq c\times max\left(\sum_{i=1}^{n}y_{i,j}\right),j\in J$$

where *x*_*i*,*j*_ is the value of deadwood availability and *y*_*i*,*j*_ the value of harvest income in stand *i* given the management regime *j*, *n* is the number of stands in the planning region, *J* is the set of all possible management regimes, and *c* is the required level of the constraint that ranges from 0 to 1 by 0.02. The optimization problem was formulated and solved using the IBM ILOG CPLEX optimizer version 12.8.0 (https://www.ibm.com/developerworks/downloads/ws/ilogcplex/).

The optimization problem was solved for each planning region separately after which the levels of the objectives were summed across the planning regions to calculate the overall outcome for that delineation of the planning regions. These outcomes were then compared with each other to reveal whether better solutions to the conflict could be found by increasing the size of the planning regions. The outcomes were compared in terms of i) the mean value of deadwood availability (m^3^/ha) and ii) the percentage of total forest area where mean deadwood availability was greater than 20 m^3^/ha. The latter measure is based on the proposed minimum of 20 m^3^/ha of deadwood as a threshold level for the occurrence of rare saproxylic polypore species [43,44]. Polypores are a species group with a high proportion of red-listed species in Finland (approximately 40% of assessed species [5]), and are considered as indicators of forest naturalness and biodiversity [43]. We note that this criterion is somewhat stricter in our system where the volume of deadwood is multiplied by its diversity. In addition, the variation in stand-level values of deadwood availability was examined to explore the implications of management optimization at different scales to the distribution and evenness of deadwood resources. High variation across stands may be caused by a trade-off between habitat quality and habitat continuity that we hypothesize is more pronounced at larger spatial scales. Stand-level variation was quantified with the coefficient of variation (the ratio of standard deviation to mean). We also recorded the distribution of the alternative management regimes that was identified as optimal at each point of the production possibility frontier and compared these distributions across scales.

## Results

### Trade-off between harvest income and deadwood availability

**Fig 2:**
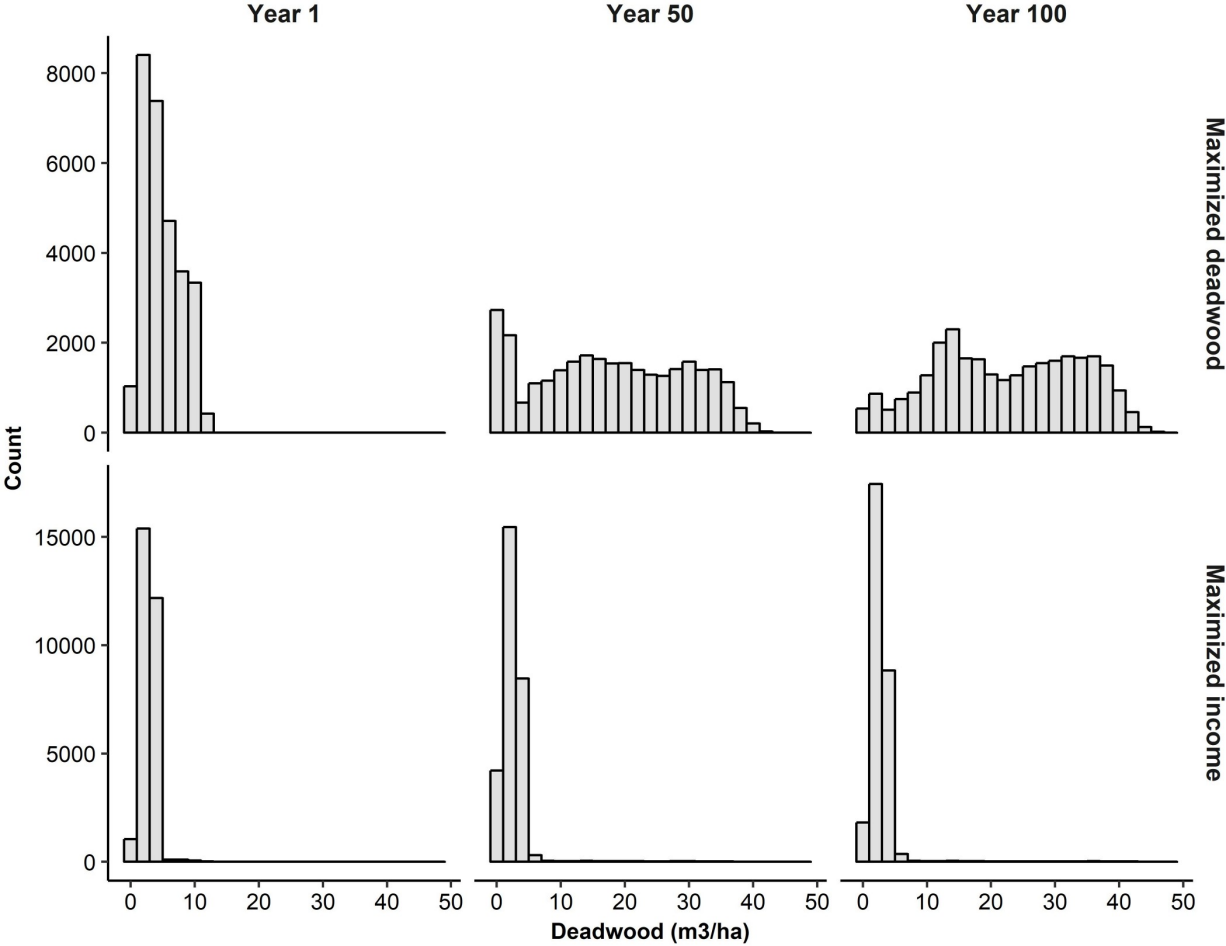
Development of deadwood availability. Distributions of stand-level values of deadwood availability (m^3^ per ha) at the beginning, halfway, and end of the 100-year simulation period when deadwood availability was maximized without a harvest income constraint (top row) and when maximal harvest income was required (bottom row). Note that the x-axis are the same in all plots, but the y-axis differ between the top and bottom row.

The maximal net present value of harvest income from the study area was 433 million euros, or 9,800 euros per ha, and the maximal level of deadwood availability was 16.0 m^3^ per ha (temporal mean over the 100-year simulation period). When harvest income was required to reach its maximal potential, mean deadwood availability was only 2.6 m^3^ per ha. Without the harvest income constraint, deadwood availability increased over time: at the beginning of the simulations, mean deadwood availability was 4.9 m^3^ per ha, halfway through the simulation period 17.2 m^3^ per ha, and at the end of the simulation period 22.0 m^3^ per ha (Fig 2). With maximized harvest income, there was little temporal change in deadwood availability as its respective mean values were 2.9 m^3^ per ha, 2.4 m^3^ per ha, and 2.6 m^3^ per ha (Fig 2). Maximal deadwood availability was reached only when the required level of harvest income was 6% or less of its maximum. At best, the two objectives could simultaneously reach around 62% (‘small holding’), 63% (‘large holding’), or 64% (‘watershed’, ‘region’) of their maximal values.

**Fig 3:**
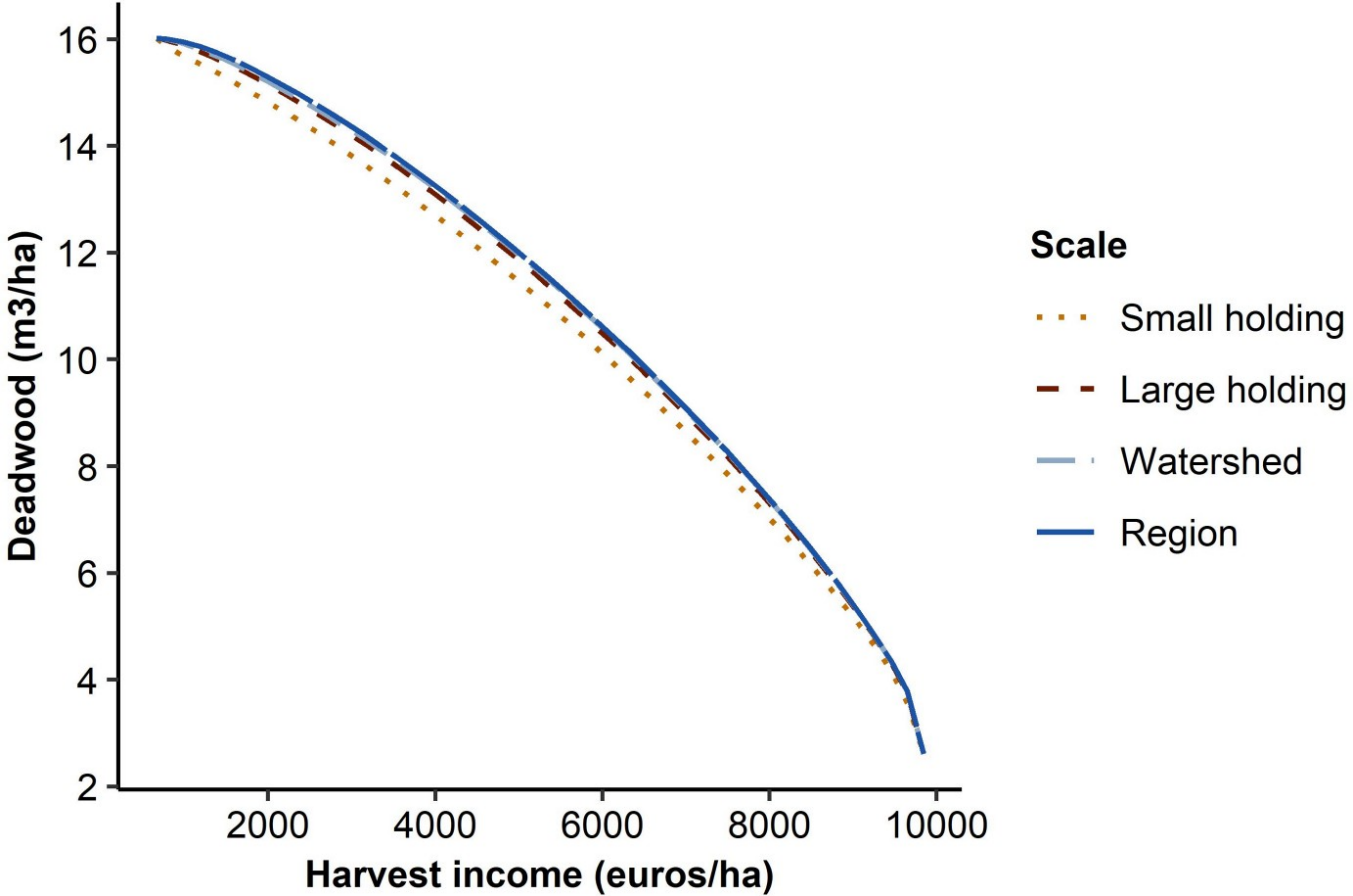
Trade-off between harvest income and deadwood availability. Production possibility frontiers illustrating the trade-off between harvest income and deadwood availability when forest management was optimized at different spatial scales.

**Fig 4:**
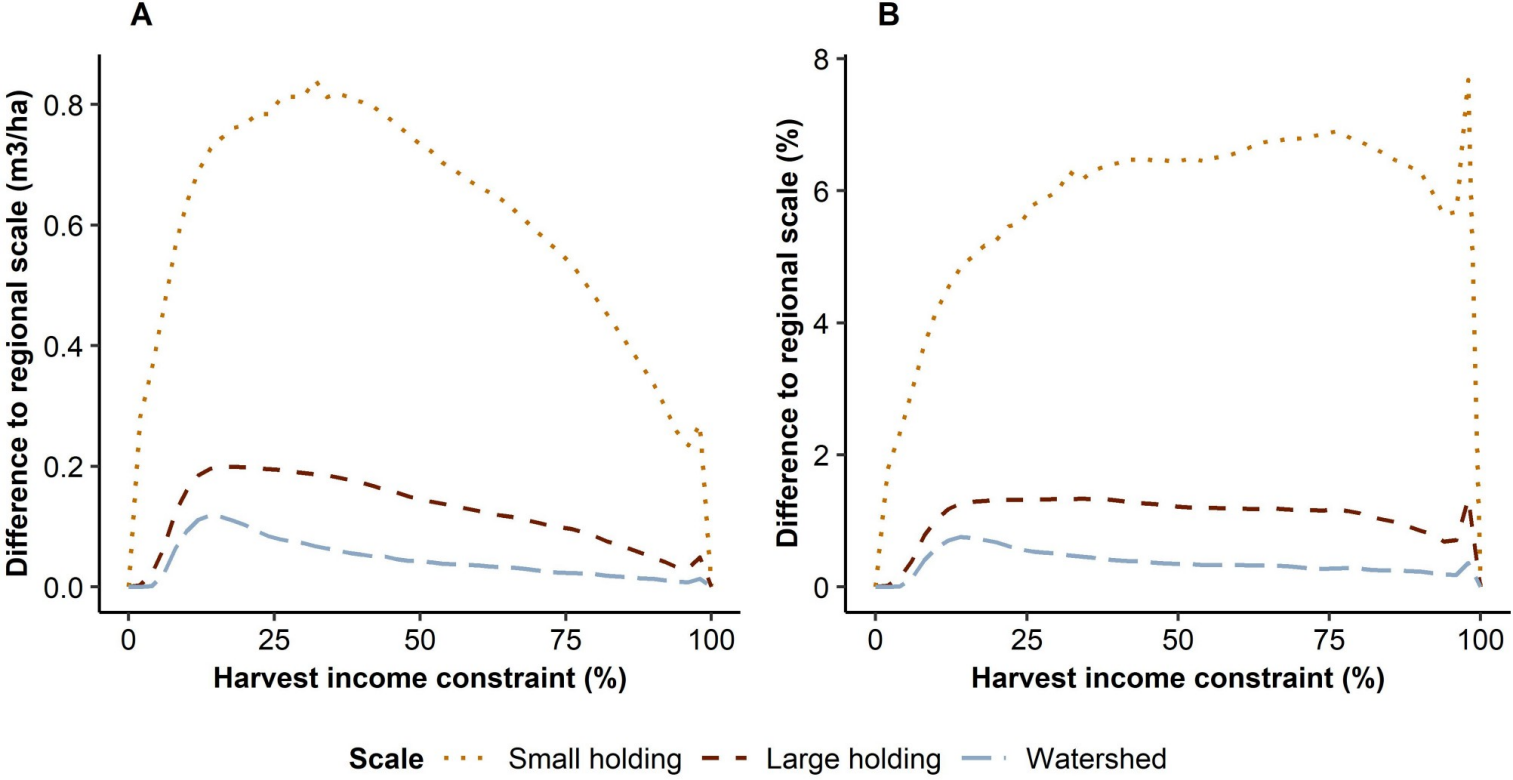
Differences in deadwood availability among scales. Improvement gained from increasing the scale of management optimization from the three smaller scales to the regional scale expressed as the absolute (panel A) and relative (panel B) difference in deadwood availability.

As expected, higher levels of the two objectives could be reached simultaneously as the size of the planning regions was increased. This was illustrated by the shapes of the trade-off curves (Fig 3). The curve was the further from origin the larger the scale of the planning regions was, indicating more efficient solutions to the conflict between the two objectives. However, the differences between the planning region delineations were quite small. In particular, management optimization at the three largest scales (‘large holding’, ‘watershed’, and ‘region’) led to nearly overlapping outcomes (Fig 3). Indeed, the differences in the outcomes between the ‘small holding’ scale and the ‘large holding’ scale were larger than the differences between the ‘large holding’ scale and the two largest scales (Fig 4). The largest absolute differences in deadwood availability between the planning region delineations were found at low-to-medium harvest income requirements, peaking at a 0.84 m^3^/ha difference between the ‘small holding’ scale and the regional scale at the harvest income requirement of 32% (Fig 4A). In relative terms, the differences were the highest at high harvest income requirements and peaked at 8% between the same ‘small holding’ scale and the regional scale at the harvest income requirement of 98% (Fig 4B). The differences between the ‘large holding’ scale and the regional scale were at most 0.20 m^3^/ha, or 1%.

**Fig 5:**
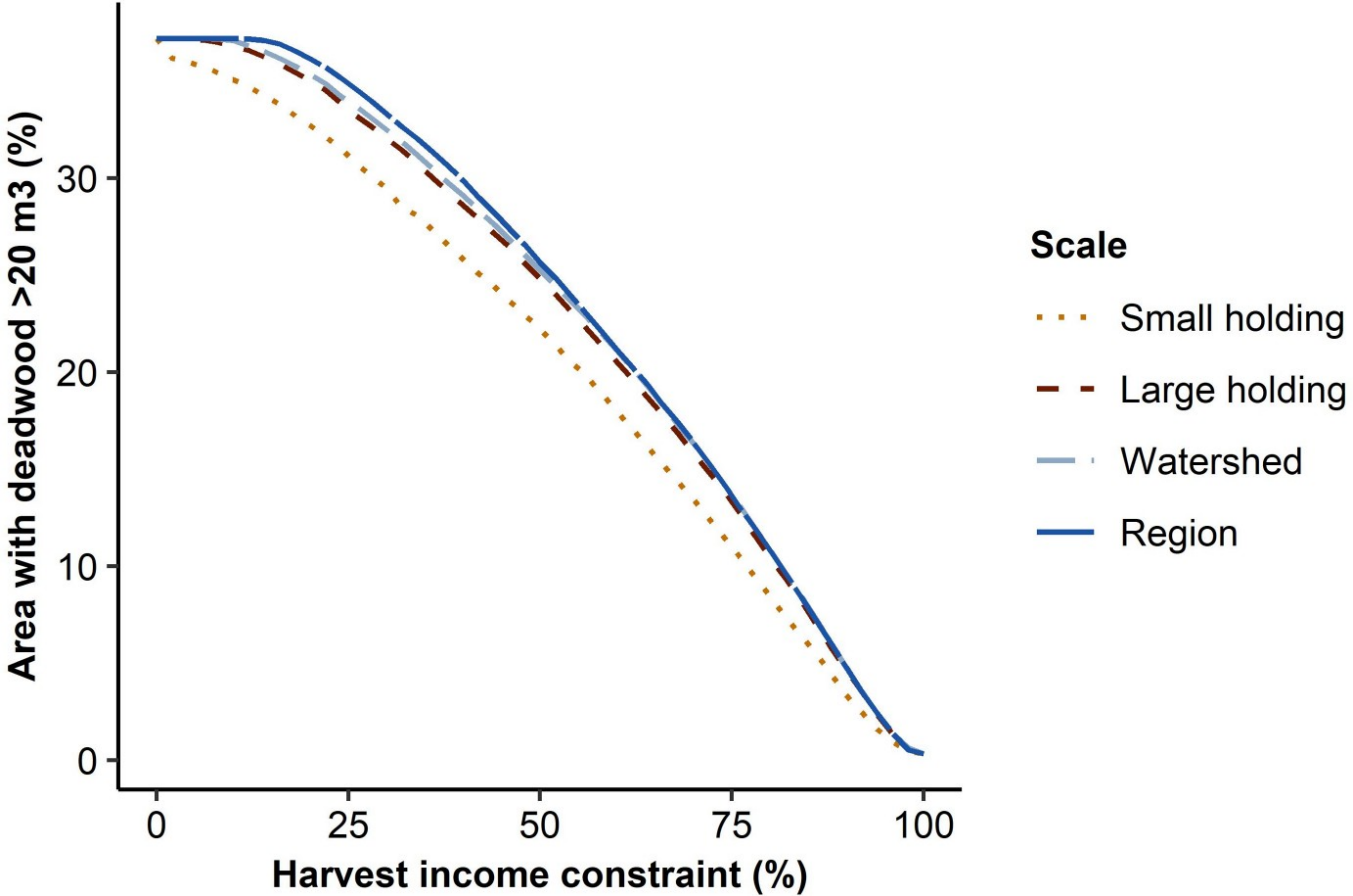
Proportion of forest area with high deadwood availability. Proportion of forest area with deadwood availability greater than 20 m^3^/ha at different harvest income requirements and under forest management optimized at different spatial scales.

The proportion of forest area with deadwood availability greater than 20 m^3^/ha was the higher the smaller the harvest income requirement was, and at most reached 37% (Fig 5). It was also the higher the larger the planning regions were (Fig 5). Other than close to the extremes of the harvest income requirement (100% or 0%), the proportion was consistently 2–4 percentage points lower at the ‘small holding’ scale than at the three larger scales, and 0.5–1 percentage points lower at the ‘large holding’ scale than at the two larger scales.

Measured with the coefficient of variation, among-stand variation in deadwood availability was higher at smaller than larger spatial scales except at harvest income requirements close to maximum (Table 3). Overall, the differences in the coefficients of variation between planning region delineations where quite small (a few percentage points) and, again, the largest between the ‘small holding’ scale and the three larger scales (Table 3).

**Table 3:** Among-stand variation in deadwood availability. Coefficients of variation in the values of deadwood availability across stands at different harvest income requirements (90%, 70%, 50%, 30%, or 10% of its maximal value).

Scale	Income 90%	Income 70%	Income 50%	Income 30%	Income 10%
Small holding	1.01	0.91	0.80	0.71	0.64
Large holding	1.05	0.91	0.78	0.68	0.60
Watershed	1.04	0.90	0.77	0.67	0.60
Region	1.04	0.90	0.77	0.67	0.59

### Optimal management plans

**Fig 6:**
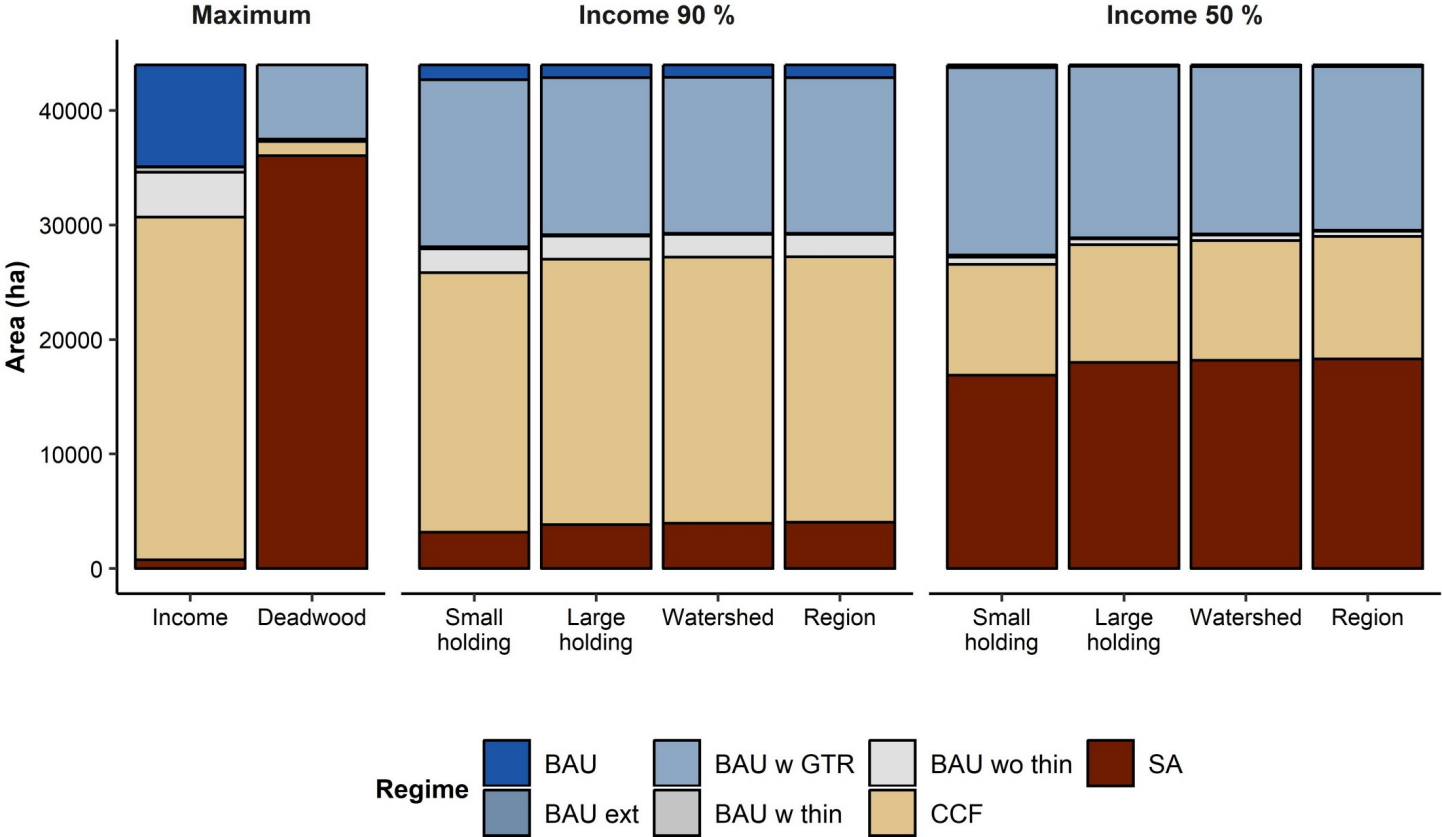
Distributions of management regimes. The distributions of management regimes that maximize the two objectives (‘Income’ and ‘Deadwood’) or provide the optimal outcomes at harvest income requirements of 90% and 50% when management regime allocation is optimized at different spatial scales (‘Small hold.’ for the ‘small holding’ scale and ‘Large hold.’ for the ‘large holding’ scale). For visual clarity, the 16 modified versions of ‘business-as-usual’ management (see Methods) have been grouped into 4 categories based on their defining features (extended rotation time, green tree retention, thinnings before final felling, or no thinnings). The abbreviations in the legend refer to: BAU—business-as-usual; BAU ext—business-as-usual with extended rotation time; BAU w GTR—business-as-usual with green tree retention; BAU w thin—business-as-usual with thinning before final felling; BAU wo thin—business-as-usual without thinnings; CCF—continuous cover forestry; and SA—set-aside.

The optimal distributions of the management regimes were clearly different for maximizing either one of the objectives and for aiming at high levels of both (Fig 6). When maximal harvest income was targeted, majority of the stands were assigned the continuous cover forestry regime (CCF; 68% of forest area) followed by ‘business-as-usual’ rotation forestry (BAU; 20% of area) and ‘business-as-usual’ forestry without thinnings (BAU wo thin; 9% of area). When deadwood availability was maximized, most of the forest area was set aside (SA; 82% of forest area) and the second most common regime was ‘business-as-usual’ forestry with green tree retention (BAU w GTR; 15% of area). Management balancing the two objectives at different required levels of harvest income was a combination of these five regimes with the exact proportions depending on the scale of the analysis, albeit also here the differences between the three largest scales were small (Fig 6). Overall, as the harvest income requirement was reduced, the proportion of forest area managed with the deadwood-favoring regimes (SA and BAU w GTR) increased. The larger the size of the planning regions was, the more SA and CCF were favored over BAU w GTR (Fig 6).

## Discussion

This study aimed to resolve the question of how large forest areas are required for forest management optimization to be effective in reconciling the conflicting objectives of timber harvesting and biodiversity conservation in Finland, a case exemplifying the wide-spread conflicts between resource production and conservation. Using discounted harvest income and availability of deadwood resources as indicators of forestry and conservation objectives, we optimized the allocation of forest management regimes at increasing spatial scales and compared the achievable outcomes. Our results show that increasing the size of the planning regions within which management is optimized does produce higher simultaneous levels of the two objectives, but the effect is rather small. When the required level of harvest income was kept constant but the spatial scale was changed, the differences in achievable levels of deadwood were most often of the magnitude of a few percentages. Similarly, the differences among the scales in management combinations identified as optimal were minor.

Currently, the typical amount of deadwood in Finnish production forests is only a few cubic meters per hectare. According to Finnish forestry statistics, the average amount of deadwood is 3–4 m^3^ per ha in southern and central Finland [28]. Long-term natural development of deadwood volume in such initially deadwood-poor forests has been predicted by for example Hekkala et al. [45] to be slow, taking several decades to reach values over 20 m^3^ per ha. The mean values of deadwood availability predicted by our results–from approximately 3 m^3^ per ha under timber-intensive management to 22 m^3^ per ha after 100 years of mainly set-aside management–were thus similar to these previously published values. However, we note that the results are not entirely comparable as our values of deadwood availability were calculated by multiplying total deadwood volume with deadwood diversity.

The amounts of harvested timber and deadwood in the forest are directly linked, as all wood removed from the forest decreases the deadwood pool [6]. Likewise, wood left to decay in the forest is wood that cannot be harvested. Our framework of forest growth simulation and multi-objective management optimization demonstrates this as a trade-off between income from timber harvesting and deadwood availability. When either one of harvest income or deadwood availability were maximized, the other was far from its potential maximal level, and the two could simultaneously reach 62–64% of their maximal levels, depending on the spatial scale of the management optimization. Earlier studies utilizing a similar framework have shown a trade-off between timber harvesting and deadwood availability at the stand scale [46] as well as at a large landscape scale [22]. In particular, Triviño et al. [22] showed the utility of landscape-scale forest management optimization to mitigate the trade-off and thus recommended landscape-scale forest management planning as a way to reconcile conflicting management objectives. The current study adds to this knowledge by showing that better solutions to the trade-off can indeed be found by optimizing management over larger forest areas. However, our results indicate that management optimization over comparatively small areas (some hundreds of hectares) already captures most of the utility of the optimization process. Whether measured as mean deadwood availability or proportion of high-quality habitat (deadwood availability greater than 20 m^3^/ha), the differences between the planning region delineations were small.

On the contrary to the value across stands and to our expectation, we found the variation in deadwood availability among stands to be lower at larger scales of management optimization. Our expectation was that when compromised with income from timber harvests, higher overall levels of deadwood availability are achieved by prioritizing it more in some stands and less in others, and these extremes become more common when more stands are included within the planning region (corresponding to increasingly efficient ‘land-sparing’ type management [47]). Our result suggests that management planning over larger areas improves not only the overall level of deadwood availability but also its evenness in space, as that is likely linked to among-stand variation. Then again, like the differences in deadwood availability itself, also the differences in among-stand variation were very small. Moreover, we note that the coefficient of variation does not illustrate the spatial arrangement of deadwood resources (see further discussion below).

The two objectives included in this study, income from timber harvesting and deadwood availability, were favored by different types of stand management. Using forest growth simulations, earlier studies have found that refraining from all timber harvests is the most effective way to increase the amount of deadwood in forests as compared with a range of management regimes with varying schedules and levels of harvesting [22,46]. In the current study also, decreasing the harvest income requirement led to an increase in area set aside from harvesting. In addition to permanent set-aside, ‘business-as-usual’ forestry with green tree retention was increasingly favored as the harvest income requirement was lowered. This regime followed the current Finnish even-aged forestry recommendations except for an increased level of tree retention at final felling. High level of tree retention can contribute to maintaining a continuous supply of deadwood in the years following final felling [48]. When maximizing harvest income, most of the forest was managed under the continuous cover forestry regime. Continuous cover forestry has been found to be economically optimal in many cases [49] and has also been suggested to have the potential to produce higher deadwood volumes than rotation forestry [50]. Our results, however, found continuous cover forestry to be applied the less the lower the harvest income constraint was. This result may be a product of the exact formulation of the continuous cover forestry regime in the forest growth simulations, and the regime’s ability to contribute to a high deadwood supply could be improved by explicit retention of large trees [20]. We note that none of the management alternatives included in this study involved active deadwood creation, which may considerably increase deadwood availability even as compared to set-aside without restoration [45]. Including such management alternatives likely would have led to higher maximal levels of deadwood availability that could be achieved over the simulation time and potentially to an even stronger conflict with timber harvesting.

In this study we used total deadwood volume multiplied by the diversity of deadwood types as an indicator of the forest biodiversity conservation objective. It should be noted that factors other than deadwood volume and diversity can significantly impact the occurrence of deadwood dependent taxa, such as microclimate, the associated species community, and the position, spatial arrangement, and temporal continuity of the resource [6,51–53]. Therefore, it may be necessary to incorporate also these factors into management planning in order to ensure that management actions actually promote the desired deadwood-dependent taxa [31]. This would unavoidably complicate the management optimization process, as well as potentially reveal the conflict between timber harvesting and biodiversity conservation to be more severe than indicated here. However, knowledge about the responses of deadwood-dependent taxa to factors more complicated than deadwood amount and diversity, such as its spatial and temporal patterns, is still insufficient to reliably link them with forest management practices [54]. Another factor we were not able to account for in the analysis was climate change, which will likely influence deadwood dynamics and associated biodiversity in Finnish forests in the future by altering the rates of tree growth, mortality, and decomposition [55].

Overall, our findings are similar to those of our earlier study [27], where we examined the effects of planning region size on the trade-off between timber harvesting and carbon storage and on the optimal forest management to reconcile them, and found the effects to be small beyond the ‘large holding’ scale. Clearly, there is variation among stands that makes it valuable to optimize management allocation across multiple stands, be it to reconcile timber harvesting and deadwood availability or timber harvesting and carbon storage, but the amount of this variation is not significantly increased beyond scales of approximately 100 stands or 200 ha (corresponding to the ‘large holding’ scale). The results of both studies suggest that it is worthwhile to conduct multi-objective forest management even at relatively small spatial scales. We think that this message could motivate individual forest owners to purposefully target multiple ecosystem services and conservation objectives within their holdings, as well as public agencies to offer support for such efforts. Then again, we repeat the above caveat that deadwood availability as measured in the current study as a static, stand-level quantity is a rather simple biodiversity indicator. If conservation activities target specific taxa, it is recommendable to take into account deadwood dynamics at the relevant spatial and temporal scales as realistically as possible to ensure their efficiency [31]. Methods to aid in this task should be further developed. In addition, we encourage studies similar to ours to be conducted in other ecosystems and land-use contexts to guide sustainable ecosystem management and conservation actions at appropriate spatial scales. While land-use optimization is a common theme in conservation research, the impacts of spatial scales and delineations remain little explored.

Forestry planning and forest management at large spatial scales can be difficult in contexts such as Finnish production forests, where small-scale private ownerships are prevalent [28]. This can be seen as a hindrance to the effective use of available planning tools such as multi-objective optimization methods that could help balance conflicting management objectives. Our study indicates that the conflict between timber harvesting and the availability of deadwood resources for wildlife is only marginally easier to mitigate at large spatial scales than at small ownership scales. The results are, on one hand, encouraging in that they suggest that close to best possible compromises for the two objectives can be even achieved even in small-scale forestry with careful planning. Mechanisms already exist to encourage forest owners to pursue conservation values within their lands and get recognition or compensation for it. The Forest Biodiversity Programme for Southern Finland METSO offers forest owners financial compensation for the voluntary protection of valuable habitats on their land. The certification schemes PEFC and FSC require setting aside a proportion of the forest land, and the group certification option within these schemes could be utilized to enable planning over multiple holdings. On the other hand, the results can be interpreted as discouraging for the efficient reconciliation of forestry and conservation: even when the required level of economic benefit was low, the levels of deadwood available in the forest (at most a mean of 16.0 m^3^/ha) were far from what is found in natural forests (up to 90 m^3^/ha [6]), and increasing the spatial scale of management optimization did little to change this fact. Setting aside forest land as part of certification requirements or voluntary agreements is of limited benefit if deadwood availability is low and takes decades to recover in even the most valuable stands. The conflict between economic gains and deadwood availability is inherent to modern forestry and solving it is urgent [19]. However, it appears that it may be impossible without novel stand management practices, active deadwood restoration, or a re-defining of forestry objectives.
